# A hierarchical (multicomponent) model of in-group identification: adaptation of a measure to the Brazilian context

**DOI:** 10.1186/s41155-019-0131-6

**Published:** 2019-10-12

**Authors:** Luana Elayne Cunha de Souza, Tiago Jessé Souza de Lima, Luciana Maria Maia, Ana Beatriz Gomes Fontenele, Samuel Lincoln Bezerra Lins

**Affiliations:** 1 Universidade de Fortaleza, Av. Washington Soares, 1321, Sala N-13, Edson Queiroz, Fortaleza, Ceará 60811-905 Brazil; 2 Universidade do Porto, Porto, Portugal

**Keywords:** Identity, In-group identification, Social identity, Group, Self-stereotyping

## Abstract

The aim of this study is to adapt the multidimensional in-group identification scale (MGIS) to the Brazilian context by gathering evidence of its psychometric properties. A total of 663 people from two samples participated in the study. In sample 1, we measured the identification of Brazilians with the region of the country where they live. In sample 2, we measured the identification of students with the university which they attend. Confirmatory factor analyses were performed on both samples to compare the models previously proposed by the original authors of the measure. The obtained results confirmed the validity of the hierarchical and multidimensional factor structure proposed by the original authors. The scale proposed here can be used to measure multiple dimensions of in-group identification in Brazil.

## Background

Humans are social beings that, when defining themselves as individuals, do so by referring to the social groups to which they belong. According to the social identity theory (Tajfel, 1978; Tajfel & Turner, 1986) and the theory of self-categorization (Turner, Hogg, Oakes, Reicher, & Wetherell, 1987), in-group identification is an important part of an individual’s self-concept that affects attitudes and behaviors. Awareness of belonging to a social group leads the individual to think and behave in the way the members of the group do.

Thus, group membership has serious implications for experience and behavior (for a review, see Tajfel & Turner, 1979). It also has psychological and social consequences for individuals (for a review, see Ellemers, Spears, & Doosje, 1999). In-group identification is an indispensable construct to understand intra and intergroup dynamics (Leach et al., 2008).

Although most studies treat identification as a general connection to the in-group and operationalize it as a one-dimensional construct (Postmes, Haslam, & Jans, 2013; Reysen, Katzarska-Miller, Nesbit, & Pierce, 2013; Wachelke, 2012), this approach seems to be inadequate conceptually and empirically (for reviews, see Ashmore, Deaux, & McLaughlin-Volpe, 2004; Leach et al., 2008; Sellers, Smith, Shelton, Rowley, & Chavous, 1998). As a solution, some studies have identified components of in-group identification such as “self-categorization,” “affective commitment,” and “centrality” (e.g., Cameron, 2004; Ellemers, Kortekaas, & Ouwerkerk, 1999; Jackson, 2002; Luhtanen & Crocker, 1992). However, until the work of Leach et al. (2008), there was a little agreement about the precise number and nature of these components, as well as to how they fit into a general conceptual model.

To address this issue, based on a review of previous works, Leach et al. (2008) developed a hierarchical and multidimensional model of in-group identification. The authors reviewed previous multidimensional methods and identified five distinct components of in-group identification: *self-stereotyping*, *in-group homogeneity*, *solidarity*, *satisfaction*, and *centrality*.

Regarding *self-stereotyping*, the identification with a group presupposes a self-categorization that includes the individual in the group (Tajfel, 1978; Turner et al., 1987). However, identification with a group means more than a simple inclusion in a group (Tajfel, 1978). The discussion of Campbell (1958), Lewin (1948), and Turner et al. (1987) which applies the theory of self-categorization supports this dimension. Individuals may self-stereotype by perceiving themselves as similar to prototypical members of the in-group (for a review, see Oakes, Haslam, & Turner, 1994).

*In-group homogeneity* refers to the degree to which individuals perceive members of their group share common aspects (e.g., Doosje, Ellemers, & Spears, 1995; Lickel et al., 2000). This sharing makes the group relatively homogeneous (Oakes et al., 1994; Simon, 1992). The perception of in-group homogeneity establishes the group as a coherent social entity. Thus, in-group homogeneity is associated with the perception that the in-group is different from out-groups (Oakes et al., 1994; Turner et al., 1987). Although the perception of in-group homogeneity has been studied extensively, no multicomponent approach to prior in-group identification has specified it as a component.

In regard to *satisfaction*, the identification of an individual with his or her group is eminently shown by the positive feeling of belonging to a particular group (Tajfel, 1978; Tajfel & Turner, 1979). However, the conceptualization and measurement of such satisfaction vary according to previous multicomponent methods. For example, several researchers have combined positive and negative feelings about the group into a single component, yet such affections tend to be independent. Satisfaction is especially associated with maintaining a positive in-group assessment (for a review, see Ashmore et al., 2004). Therefore, satisfaction may lead people to minimize negative events or resist negative portrayals of the in-group in an attempt to maintain their satisfaction with their in-group.

As for *solidarity*, the early social psychological notions of in-group identification emphasized the solidarity component (for a review, see Cartwright & Zander, 1968). For example, Lewin (1948) suggested that people that most strongly identify with the in-group are more inclined to feel a psychological bond with its members. A recent work on the tradition of social identity emphasizes a psychological and behavioral “commitment” to the group, in the same way as previous approaches do to solidarity (for a review, see Ellemers, Kortekaas, & Ouwerkerk, 1999). As solidarity is based on commitment and psychological bonds with the members of the in-group, it should be associated with a sense of belonging, a psychological attachment to the in-group, and activities coordinated with other group members (Leach et al., 2008).

Regarding *centrality*, the self-categorization theory suggests that in-group identification makes the group a central aspect of the individual’s self-concept (see Oakes et al., 1994; Turner et al., 1987). The centrality of group belonging is shown by its chronic salience as well as by the subjective importance that individuals attribute to that belonging (for reviews, see Ashmore et al., 2004; Turner et al., 1987). For Leach et al. (2008), it is likely that the centrality component of in-group identification makes individuals perceive a great threat to their group, whether real or symbolic. Since this perception of threat tends to encourage active coping, centrality may lead individuals to defend their in-group against a perceived threat. Thus, the more central the in-group, the more individuals defend this group against threats. An unimportant in-group is not worth defending.

Leach et al. (2008) reviewed previous theories and studies to identify five strictly specified components. Rather than simply contributing to the proliferation of multicomponent methods, they sought to integrate these components into a general conceptual framework. As a result, they have proposed a model with two general dimensions. They specify how the five components are related to each other.

The first second-order dimension is *self-definition*. Identifying a group in terms of self-definition should manifest itself in the individual’s perception of self as being similar to a prototype of the group. It also manifests in the perception that individuals have of the in-group in sharing common aspects. The second dimension is called *self-investment*. Identification with a group in terms of self-investment should manifest itself in positive feelings of the individuals in relation to group belonging in the sense that they have a link to the in-group, as well as the saliency and importance of belonging to that group.

Leach et al. (2008) operationalized this multidimensional hierarchical model in a measure containing 14 items. Most of these items are adaptations similar to previous measures. The authors validated their measure through seven studies using different groups (Dutch, European, and university students). The results attest that the five-component model and the two second-order factors fit the data and that the measure shows good internal consistency, construct validity, concurrent validity, and discriminant validity.

As Lovakov, Agadullina, and Osin (2015) argued, this model of in-group identification is important because it was created by articulating multiple approaches, i.e., the classical and the contemporary models of in-group identification. It specifies similarities and differences between group components. Thus, the measure based on this model can be used to study identification with different social groups.

In this regard, Lovakov et al. (2015) made a brief survey of studies in which the measure of Leach et al. (2008) was applied to evaluate identification with different groups. The authors observed that, among the main groups studied, there are ethnic, national, and racial in-groups (Danel et al., 2012; Giamo, Schmitt, & Outten, 2012; Koval, Laham, Haslam, Bastian, & Whelan, 2012; Leach, Mosquera, Vliek, & Hirt, 2010; Philpot & Hornsey, 2011; Shepherd, Spears, & Manstead, 2013; Stürmer et al., 2013; Wang, Minervino, & Cheryan, 2013), gender in-groups (Correia et al., 2012; Good, Moss-Racusin, & Sanchez, 2012; Kenny & Garcia, 2012), student in-groups (Becker, 2012; Correia et al., 2012; Cruwys et al., 2012; Leach et al., 2010), virtual in-groups (people in an online community that share the same interests) (Howard, 2014; Howard & Magee, 2013), the army (Sani, Herrera, Wakefield, Boroch, & Gulyas, 2012), an experimental in-group (Hartmann & Tanis, 2013; van Veelen, Otten, & Hansen, 2013), mental health in-groups (Gee & McGarty, 2013), and an organizational in-group (Smith, Amiot, Callan, Terry, & Smith, 2012).

However, to the best of our knowledge, the vast majority of studies using this scale have been developed in English, with the exception of the research by Danel et al. (2012), Correia et al. (2012), and La Barbera and Capone (2016), which were in Polish, Portuguese, and Italian, respectively. In addition, three scale validation studies were found, one for the Russian context (Lovakov et al., 2015), one for the Italian context (La Barbera & Capone, 2016), and another for the Portuguese context (Ramos & Alves, 2011).

Due to the importance of the topic for the understanding of a series of social phenomena, the existence of a valid and precise measurement with a consistent theoretical foundation is pertinent and necessary for the Brazilian context. In this sense, the aim of the present study is to adapt the multidimensional in-group identification scale (MGIS) proposed by Leach et al. (2008) to the Brazilian context by gathering evidence of its psychometric properties (construct validity and precision).

## Method

### Participants

The present study consisted of 663 participants from two samples. Sample 1 was composed of 146 participants from the general population. The age ranged from 18 to 60 years (*M* = 31.61, SD = 10.02). Individuals came from different regions of the country; the majority were from the Northeast (70.6%), predominantly women (61.6%), with a high educational level (48%). Sample 2 was composed of 517 university students from a private educational institution located in the Northeast of Brazil. The age ranged from 18 to 64 years (*M* = 25.3, SD = 8.1), and the majority were females (68.5%).

### Instrument

All participants filled in a questionnaire that included, in addition to sociodemographic questions, the multidimensional in-group identification scale (MGIS) proposed by Leach et al. (2008). This contains 14 items distributed into five factors. The items are answered using a seven-point scale that ranges from 1 (totally disagree) to 7 (totally agree). A back-translation process was used. First, the items were translated from English into Portuguese by two bilingual PhD researchers, and after, the items were translated from Portuguese into English again by two different bilingual PhD researches to ensure translation equivalency. The translators decided that there was no appreciable difference between the original and back-translated English versions. We created two versions of the scale for each in-group identification: region of group belonging in Brazil (sample 1) and university (sample 2). For the sample that answered about regional identification, we previously asked which region of the country the person considers to be the one that represents her or him as a Brazilian. The present version can be read in the Appendix.

### Procedure

Participants were informed that participation in the research was voluntary and that the information collected would be treated confidentially. Data are used only for academic purposes, thus remaining anonymous. Data collection began only after acceptance by the participants. In sample 1, the data were collected using online questionnaires published in social networks. In sample 2, the data were collected through pencil and paper questionnaires in the classroom environment. Both samples were selected through a non-probabilistic sampling procedure by convenience. All answers were given individually. The procedures used for conducting this research met ethical and normative determinations that guide research with human beings. This research is in compliance with the determinations of the Resolutions no. 466/12 and 510/16 of the National Health Council (CNS). In addition, this research has the approval of the Research Ethics Committee of the University of Fortaleza (ruling no. 1.843.171).

### Data analysis

Multiple confirmatory factor analyses were performed using the software AMOS 18 considering the covariance matrix and the ML (maximum likelihood) estimation method. Missing data comprised 0.24% of observations. They were replaced by the average of answers obtained for each item.

In order to know the fitting of the proposed model and to compare it with alternative models, the following indicators were used: chi-square (*χ*^*2*^), standardized chi-square (*χ*^*2*^*/gl*), comparative fit index (*CFI*), normed fit index (*NFI*), root mean square error of approximation (*RMSEA*), Akaike information criterion (*AIC*), and expected cross-validation index (*ECVI*). These indexes have been commonly used in previous studies, and although each presents merits and limitations, they are strong indicators of model fitting to data when used together (Byrne, 2010). According to Byrne (2010), for the normalized chi-square (χ^2^/gl), values lower than five indicate an adequate fitting of the model. Values lower than three are desirable. Values of *CFI* and *NFI* above .90 indicate an acceptable fit, while values above .95 indicate a good fit. For *RMSEA*, values up to .08 indicate an acceptable fit, while values up to .06 indicate a good fit. Regarding the indexes of comparison between models (*AIC* and *ECVI*), low values indicate a model with a better fit (Byrne, 2010).

## Results

**Fig. 1:**
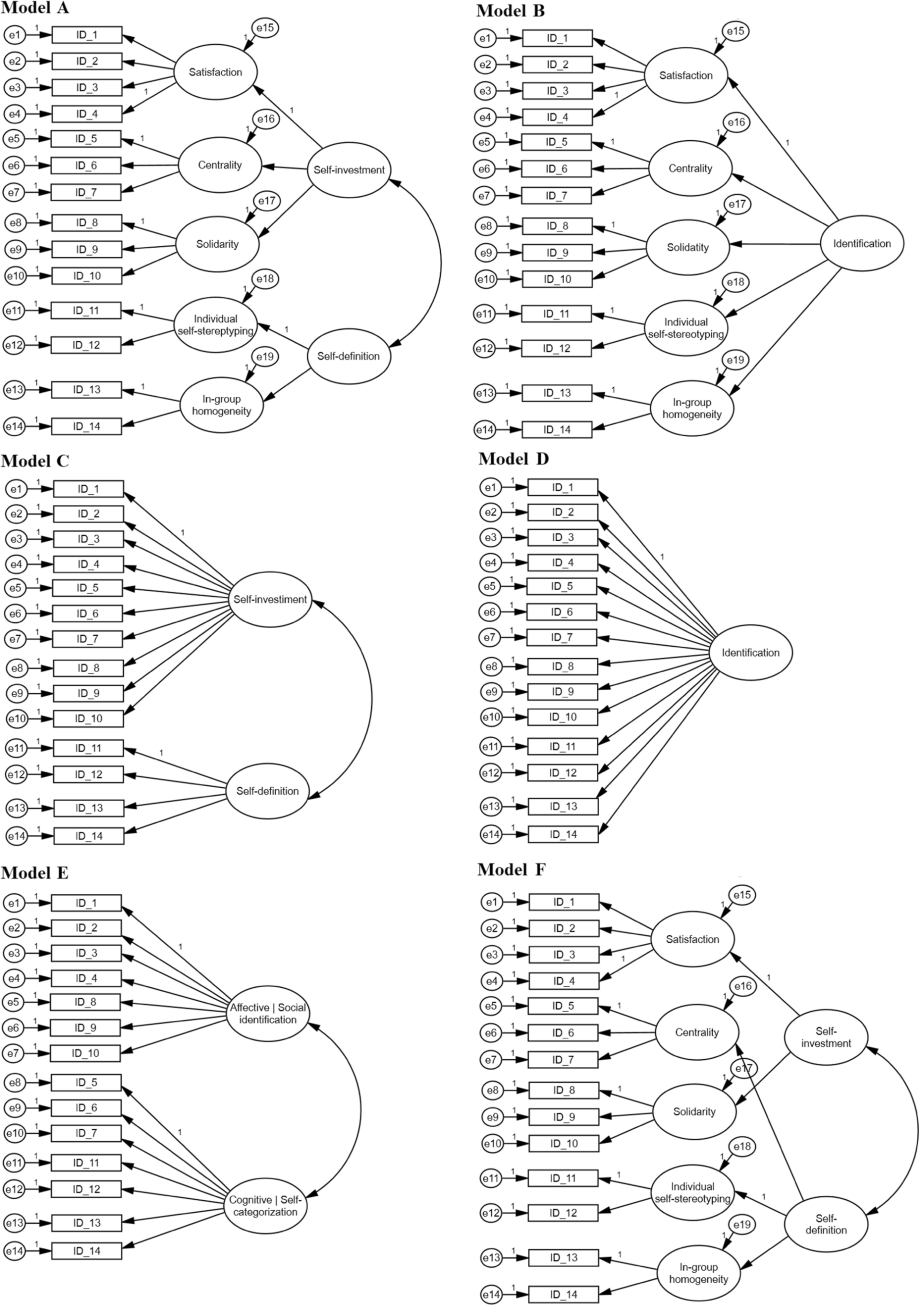
A hierarchical (multicomponent) model of in-group identification and alternative models

Initially, confirmatory factor analyses were performed to verify whether the proposed model fits the data. The model with five components (*satisfaction, centrality, solidarity, self-stereotyping,* and *in-group homogeneity*) and two second-order factors (*self-investment* and *self-definition*), as proposed by Leach et al. (2008), was analyzed for each sample separately. This model can be visualized in Fig. 1a. As can be seen in Table 1, this model had satisfactory fit indexes in both samples. The values of *NFI* and *CFI* were above .90 in sample 1 and above .95 in sample 2. These values are above the values recommended in the literature. In addition, residue values (*RMSEA*) of sample 2 are below the maximum values recommended in the literature (.08), while the residue of sample 1 is slightly above the recommended maximum value.

**Table 1:** Adjustment indicators for in-group identification models

	χ^2^ (gl)	χ^2^/gl	NFI	CFI	RMSEA [IC - 90%]	ECVI	AIC
Sample 1							
Model A	148.20 (71)	2.09	.90	.95	.087 [.067–.106]	1.49	216.2
Model B	154.79 (72)	2.15	.90	.94	.089 [.070–.108]	1.52	220.7
Model C	543.80 (76)	7.15	.65	.68	.206 [.190–.223]	4.15	601.8
Model D	638.94 (77)	8.30	.58	.61	.224 [.208–.241]	4.79	694.9
Model E	521.9 (76)	6.87	.66	.69	.201 [.185–.218]	3.99	579.8
Model F	153.1 (71)	2.16	.90	.94	.089 [.070–.109]	1.53	221.1
Sample 2							
Model A	181.5 (71)	2.56	.97	.98	.055 [.045–.065]	.48	249.5
Model B	199.1 (72)	2.77	.96	.97	.058 [.049–.068]	.51	265.1
Model C	1546.8 (76)	20.3	.70	.71	.194 [.185–.202]	3.11	1604.8
Model D	2074.8 (77)	26.9	.60	.61	.224 [.216–.233]	4.13	2130.8
Model E	1768.3 (76)	23.3	.66	.67	.208 [.199–.216]	3.54	1826.3
Model F	199.1 (71)	2.8	.96	.97	.059 [.050–.069]	.52	267.1

**Fig. 2:**
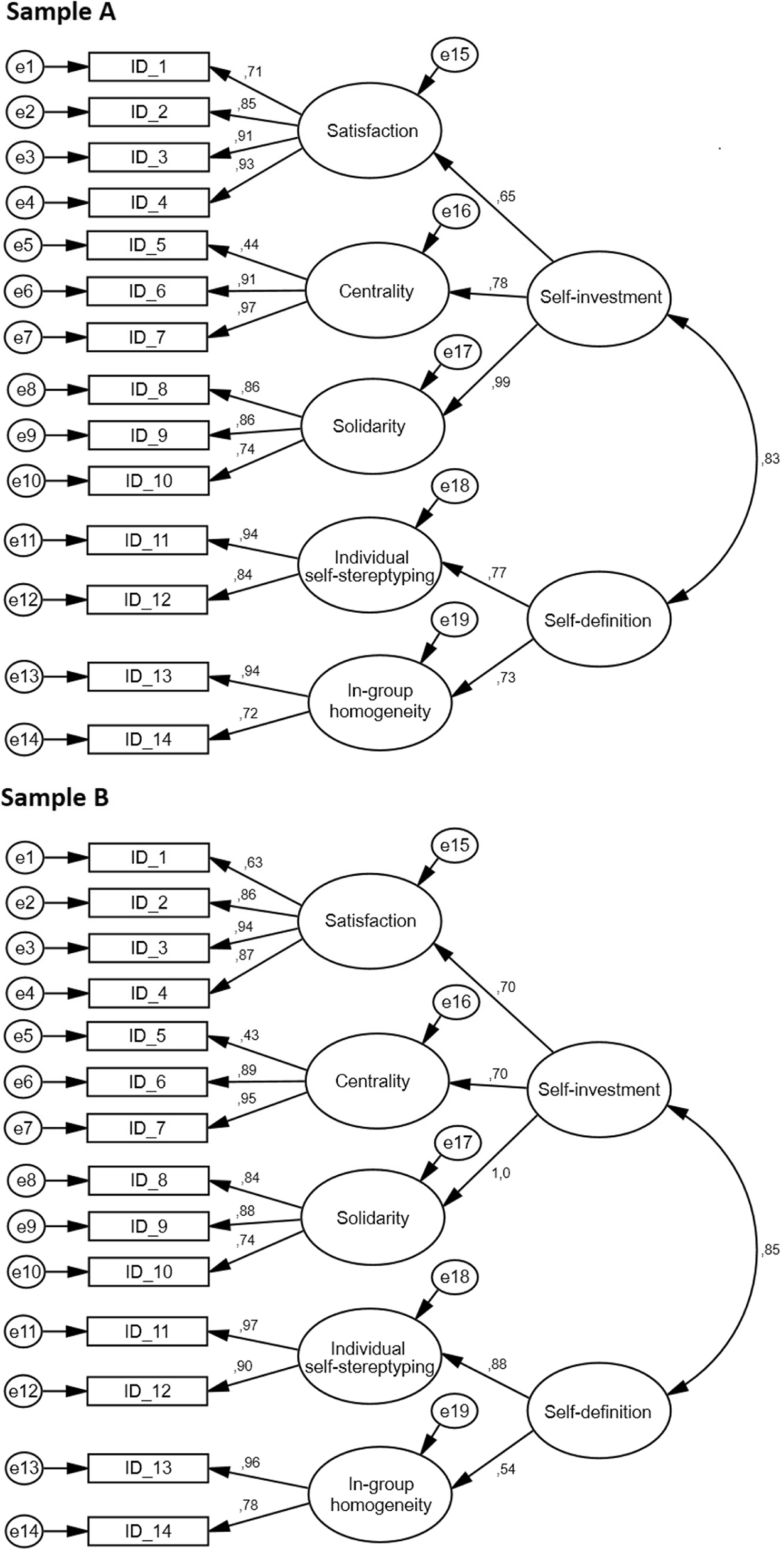
A hierarchical (multicomponent) model of in-group identification

Figure 2 presents the proposed model (model A) for both samples. In relation to sample 1, factor loadings varied between .44 and .97, all statistically significant (*p* < .001). For second-order factors, the factor loadings were above .65. All were statistically significant (*p <* .001). Both second-order factors presented a correlation of .83 (*p* < .001). In relation to sample 2, factor loadings varied between .63 and .97, all statistically significant (*p* < .001). Similarly, the factor loadings of second-order factors were above .54. All were statistically significant (*p <* .001). Both second-order factors presented a correlation of .83 (*p* < .001).

Subsequently, following the same procedures of the original work of Leach et al. (2008), we compared the proposed model with five alternative models, as shown in Fig. 1: Model B (five components predicted by a second-order factor called in-group identification), model C (two-factor model with self-investment and self-definition dimensions), model D (single in-group identification model), model E (containing the original centrality factor belonging to another dimension), and model F (model with five components and two second-order factors, but the centrality factor is predicted by the self-definition dimension).

Regarding alternative factorial structures, the models B and F had satisfactory fit indexes. The results of both samples are close to those recommended in the literature and comparable to the indexes obtained by model A. On the other hand, the fit indexes of models C, D, and E reveal that these models are not suitable for describing the data. Correspondingly, models A, B, and F have a structure with five factors. In order to compare the fit obtained by these models, the ECVI and AIC indexes were used. Data indicate that the values of both indexes are lower for model A compared to models B and F for both sample 1 and sample 2. Therefore, model A explains more of the variance in the dataset compared to the other models.

Finally, the internal consistency indexes for the five components of the measurement were calculated. These results are presented in Table 2 along with descriptive statistics and correlations among components. All five MGIS components showed good to excellent internal consistency, with Cronbach’s alpha ranging from .78 to .94 in both samples (Garson, 2012). The correlations between the components are moderate. However, in both samples, the correlations are higher among components of the same dimension. Satisfaction, solidarity, and centrality have greater correlations with each other rather than with self-stereotyping and in-group homogeneity. This is also the case for the latter two components in the self-definition dimension. These results, together with the results of confirmatory factor analyses, support the hierarchical conceptualization proposed by Leach et al. (2008). Thus, we can attest to the factorial validity of MGIS for the Brazilian context.

**Table 2:** Descriptive statistics and correlations for five components of in-group identification

Component	*α*	*M*	SD	ISS	IGH	SA	SO	CE
Region of the country								
Individual self-stereotyping (ISS)	.88	4.81	1.56	–	*.51* ^*^	.44^*^	.52^*^	.48^*^
In-group homogeneity (IGH)	.80	4.81	1.42		–	.35^*^	.51^*^	.35^*^
Satisfaction (SA)	.91	5.91	1.18			–	*.63* ^*^	*.45* ^*^
Solidarity (SO)	.86	5.42	1.44				–	*.64* ^*^
Centrality (CE)	.79	4.67	1.63					–
University								
Individual self-stereotyping (ISS)	.94	4.18	1.72	–	*.43* ^*^	.47^*^	.69^*^	.42^*^
In-group homogeneity (IGH)	.85	4.41	1.55		–	.23^*^	.41^*^	.29^*^
Satisfaction (SA)	.89	4.58	1.41			–	*.62* ^*^	*.46* ^*^
Solidarity (SO)	.85	4.80	1.54				–	*.59* ^*^
Centrality (CE)	.78	3.95	1.51					–

Note: Italic correlations are those of scales that refer to the same dimension. *p ≤ .01

## Discussion

The aim of the present study was to adapt the multidimensional in-group identification scale (MGIS) proposed by Leach et al. (2008) to the Brazilian context by gathering evidence of its psychometric properties (construct validity and precision). In order to reach this goal, we applied the scale to two samples of different groups: in the first sample, we analyzed the identification of Brazilians with their region in the country; in the second sample, we analyzed the identification of students with the university they attended. From these data, we can make some considerations.

The first consideration is that our results consistently show in both samples that the multidimensional and hierarchical structure proposed by Leach et al. (2008), in which in-group identification is organized into five components predicted by two second-order factors, fits empirical data. Thus, we can state that the proposed theoretical model is replicated in the Brazilian context. We can also state that this model has been supported across several cultures, since there are studies demonstrating that it works for the Dutch (Leach et al., 2008), North American (Howard & Magee, 2013), Russian (Lovakov et al., 2015), Italian (La Barbera & Capone, 2016), and Portuguese contexts (Ramos & Alves, 2011). Nevertheless, there is a need for further research to verify the validity of this model in the Eastern context.

The second consideration is that the Brazilian version of MGIS is suitable for different membership groups. In this sense, future research on the Brazilian context can use the scale to measure the different facets of in-group identification with different in-groups. However, one limitation of the present study was not to test the different types of model invariance in the groups analyzed. Thus, it is expected that, in future research, we will investigate if the model is invariant between different groups of belonging, as was done by Lovakov et al. (2015).

Thirdly, the Brazilian version of MGIS showed excellent internal consistency indexes for all measurement components. In addition, correlation coefficients were moderate. This suggests that multicollinearity is not an issue and that each component evaluates different aspects of in-group identification. In addition, the highest correlation coefficients occur precisely among components belonging to the same second-order dimension, which shows that the proposed hierarchical structure is relevant.

In addition to our conclusions, we must consider that this study is not free of limitations. Although we gathered evidence of psychometric properties (construct validity and internal consistency) of the MGIS, we did not analyze other important types of measurement validity. In this sense, future studies should focus on gathering complementary evidence of convergent and discriminant measurement validity. Moreover, our correlational design did not allow us to analyze other indicators of internal consistency. In this respect, future studies adopting a longitudinal design should perform a test-retest of the measurement. Finally, we also recognize that analyzing MGIS using only two groups of belonging is not enough to ensure that the scale can be used for other in-groups. In this sense, future studies should investigate the replicability of this factorial structure with other in-groups, such as racial, religious, political, and gender in-groups.

## Conclusion

In conclusion, this study contributes to increase knowledge in a research area considered of great importance within Social Psychology but that has been little explored by Brazilian studies (Lima, Silva, Carvalho, & Farias, 2017; Martins, Lima, & Santos, 2018). The theory of social identity has been one of the main methods of explanation for contemporary intergroup phenomena such as prejudice against social minorities. However, this area has yet to be fully explored in Brazilian studies. Due to the fact that researchers can now count on a valid and accurate measurement that addresses the various facets of in-group identification, the role of in-group identification is likely to become more evident in future research.

## Data Availability

The datasets generated and/or analyzed during the current study are available in the Open Science Framework repository, https://osf.io/7z4at/.

## Appendix

**Table 3:** Brazilian version of the multidimensional in-group identification scale (MGIS)

1. Eu acho que as pessoas do [endogrupo] têm muito do que se orgulhar.	
2. É muito bom ser do [endogrupo].	
3. Eu me sinto bem em ser do [endogrupo].	
4. Eu sou feliz por ser do [endogrupo].	
5. Muitas vezes eu paro para pensar no fato de que sou uma pessoa do [endogrupo].	
6. Ser do [endogrupo] é uma parte importante de como eu me defino.	
7. Ser do [endogrupo] é uma parte importante de como eu me vejo.	
8. Eu sinto que tenho um vínculo com as pessoas do [endogrupo].	
9. Eu sinto que faço parte da comunidade de pessoas do [endogrupo].	
10. Eu me sinto comprometido com as pessoas do [endogrupo].	
11. Eu tenho muito em comum com a típica pessoa do [endogrupo].	
12. Eu sou parecido com a típica pessoa do [endogrupo].	
13. As pessoas do [endogrupo] têm muitas características em comum entre si.	
14. As pessoas do [endogrupo] são muito parecidas umas com as outras.	

## Abbreviations

AIC: Akaike information criterion
CFI: Comparative fit index
ECVI: Expected cross-validation index
MGIS: Multidimensional in-group identification Scale
NFI: Normed fit index
RMSEA: Root mean square error of approximation
